# Quantitative pupillary light reflex assessment for prognosis of carbon monoxide poisoning

**DOI:** 10.3389/fmed.2023.1105705

**Published:** 2023-02-28

**Authors:** Yong Sung Cha, Sang-Bae Ko, Tae-Hwa Go, Dong Keon Lee

**Affiliations:** ^1^Department of Emergency Medicine, Yonsei University Wonju College of Medicine, Wonju, Republic of Korea; ^2^Research Institute of Hyperbaric Medicine and Science, Yonsei University Wonju College of Medicine, Wonju, Republic of Korea; ^3^Department of Neurology, Seoul National University College of Medicine, Seoul, Republic of Korea; ^4^Department of Biostatistics and Center of Biomedical Data Science, Yonsei University Wonju College of Medicine, Wonju, Republic of Korea; ^5^Department of Emergency Medicine, Seoul National University Bundang Hospital, Seongnam, Republic of Korea

**Keywords:** carbon monoxide poisoning, prognosis, cognitive dysfunction, light reflex, hyperbaric oxygen therapy

## Abstract

**Background:**

A non-reactive pupil in standard pupillary light reflex (sPLR) is regarded as a factor predicting neurological sequelae at 1-month after carbon monoxide (CO) poisoning. An automated pupillometer is used in the intensive care unit to quantitatively assess PLR. Quantitative PLR (qPLR) was superior to sPLR using penlight for prognosis of various neurological diseases. Therefore, this study aimed to analyze whether quantitative pupillary variables (neurological Pupil index [NPi] and qPLR) are superior to sPLR in predicting 1-month neurocognitive sequelae after acute CO poisoning.

**Methods:**

We performed a prospective observational study of consecutive patients with acute CO poisoning admitted to an emergency department (ED) between August 2019 and December 2020 in a single academic medical center. sPLR and pupillometer examinations (qPLR and NPi) were performed by emergency physicians at the ED on hospital days 0–2. The lowest values among those recorded within 24 h and during the total measurement period were considered the 24-h and total lowest values, respectively. Global Deterioration Scale scores were measured at 1 month as an outcome and were dichotomized into favorable (1–4) or poor (5–7) outcomes.

**Results:**

We analyzed the data of 104 adult patients with acute CO poisoning. qPLR was significantly higher in the favorable outcome group than in the poor outcome group 24-h and total lowest values (21.2% vs. 15.0%, *p* = 0.006 and 21.0% vs. 14.8%, *p* = 0.006). qPLR <18% had fair predictive power for poor neurocognitive outcomes [area under the curve (AUC), 0.70; 95% confidence interval (0.60–0.78)]. Among the patients with decreased mental status (Glasgow Coma Scale ≤12), the power of NPi and qPLR increased [AUC, 0.72 and AUC, 0.80]. NPi < 1 and qPLR <18% showed sensitivity (9.5% vs. 76.2%) and specificity (98.8% vs. 67.5%) for the prediction of poor outcomes. qPLR was significantly superior to sPLR in predicting poor neurocognitive outcomes at 1 month after CO poisoning (*p* = 0.007).

**Conclusion:**

qPLR and NPi were superior to sPLR in terms of predicting poor neurocognitive outcomes. qPLR and NPi measured from hospital days 0–2 may be valuable in predicting neurocognitive outcome.

## 1. Introduction

Neurological complications after acute carbon monoxide (CO) poisoning can range from transient headache to permanent anoxic brain damage. Although hyperbaric oxygen therapy (HBO_2_) has been attempted to minimize neurological complications, a significant percentage of patients still experience neurocognitive sequelae after acute CO poisoning (1–3). Lack of pupillary light reflex (PLR), assessed using a penlight, is a predictor of poor neurological outcome in patients with CO poisoning (4). Given that standard PLR (sPLR) using penlight has poor inter-rater reliability, more objective and quantitative methods are required to accurately assess PLR (5, 6).

An automated pupillometer has been used in the intensive care unit to quantitatively assess the PLR. Quantitative PLR (qPLR), expressed as percentage pupillary constriction in response to a calibrated light stimulus, was superior to sPLR for prognosis of various neurological diseases, including severe stroke, traumatic brain injury, or cardiac arrest (CA) (7–10). Moreover, the Neurological Pupil index (NPi), derived from an automated pupillometer, is an accurate outcome prediction tool regardless of pupil size (9, 11–15). Therefore, we hypothesized that qPLR and NPi may be more beneficial than sPLR in predicting 1-month neurocognitive outcomes after acute CO poisoning.

## 2. Materials and methods

### 2.1. Study design and setting

**Figure 1:**
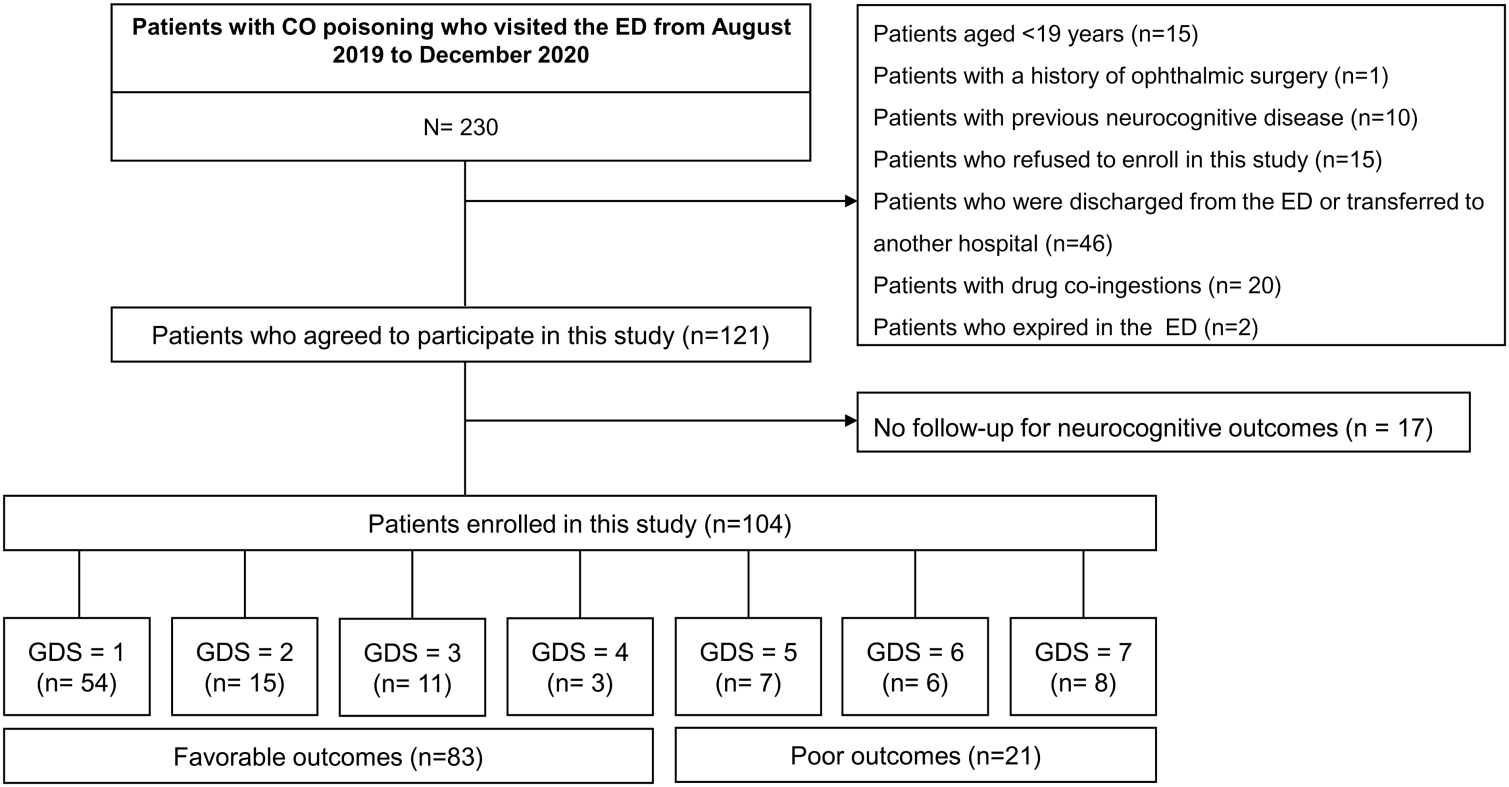
Study flow diagram. CO, carbon monoxide; ED, emergency department; GDS, global deterioration scale.

This was a prospective observational study of patients with acute CO poisoning (age ≥ 19 years) admitted to an emergency department (ED) consecutively between August 2019 and December 2020. The exclusion criteria are shown in Figure 1. Written informed consent was obtained from all patients. The study protocol complied with the ethical guidelines of the Declaration of Helsinki and was approved by the institutional review board of Wonju Severance Christian Hospital (approval number: CR319039).

CO poisoning was diagnosed based on a patient’s medical history and a measured carboxyhemoglobin (CO-Hb) level > 5% (>10% in smokers). Co-ingestion of drugs was confirmed using blood and urine tests performed by the Forensic Toxicology Division of the National Forensic Service (Wonju, Republic of Korea). Initially, all patients were treated with 100% oxygen therapy supplied through a face mask with a reservoir bag. If the patients were suspected of neurological injury, including an episode of loss of consciousness or altered mental status, cardiovascular dysfunction, severe acidosis, or CO-Hb ≥ 25%, HBO_2_ was initiated using a multi- or mono-place hyperbaric chamber (IBEX Medical Systems, Seoul, Republic of Korea) (16). Initial compression was performed up to 2.8 atmospheres absolute (ATA) for 45 min, followed by 2 ATA for 60 min. If administration of additional HBO_2_ was possible within 24 h, 2 ATA was administered for 90 min. Moreover, patients were treated with HBO_2_ after 24 h if necessary, until all symptoms had resolved.

### 2.2. Quantitative pupillary variables measured using pupillometry

Pupillary examinations were performed using an automated quantitative pupillometer (NeurOptics®, NPi®-200 pupillometer, Neuroptics Inc., Irvine, CA, United States). We evaluated qPLR, NPi, the maximum and minimum size of the pupil after light reflex, constriction velocity, maximum constriction velocity, latency, and maximum dilatation velocity. Quantitative measurements of PLR were sequentially recorded by emergency physicians from hospital day (HD) 0 to 2 after a patient’s ED visit. PLR was quantitatively measured at the time of arrival at the ED (0 h) and at the 6-, 12-, and 24-h time points on HD 0. The lowest values during a 24-h period or the lowest ones throughout the entire study periods were chosen as the 24-h lowest values or the total lowest values, respectively. If a patient was discharged before HD 2, measurements were performed until discharge. At each time point, the lowest values of the NPi and qPLR of each eye were obtained for analysis (15). sPLR was serially measured by emergency physicians in charge of the patients using a penlight. We classified the reactivity of sPLR as reactive, sluggish, or non-reactive. Non-reactive sPLR was defined when pupillary reactivity was not identified bilaterally (15).

### 2.3. Study variables and definitions

The following clinical variables were evaluated: age; sex; cause of CO poisoning (suicide attempt or accident); source of CO (non-fire or fire); maximal CO exposure time (h); concomitant use of drugs, including sedatives, neuromuscular blockers, or opioids, during admission; medical or psychiatric comorbidities; any interval of loss of consciousness; shock or intubation; Glasgow Coma Scale (GCS) scores upon arrival at the ED; need for administration of HBO_2_; duration from the time of rescue to the administration of HBO_2_ (h); and the number of times HBO_2_ was administered within 24 h after arrival at the ED and during admission. Complications (rhabdomyolysis, acute kidney injury, or pneumonia) during hospital admission were investigated, as were the following laboratory variables: blood CO-Hb levels, troponin I, bicarbonate, and lactate levels on arrival at the ED. See Supplementary Method S1 for further details of variable definitions.

We evaluated neurocognitive outcomes using the Global Deterioration Scale (GDS), ranging from 1 to 7, with higher scores indicating greater severity. The GDS was originally developed to assess patients with dementia but is frequently used to assess neurocognitive outcomes after CO poisoning (Supplementary Method S2 and Supplementary Table S1) (17–20). An independent rehabilitation physician, blinded to the pupillometer data, measured the GDS scores of the patients at 1 month. When a patient did not present to the outpatient clinic, GDS was assessed based on an interview with the patient’s guardians. GDS scores were classified as favorable (none or functionally independent; 1–4 points) or poor (bedridden; 5–7 points) outcomes, as described previously (21). We additionally evaluated delayed neuropsychiatric sequelae (DNS), a delayed onset of neurocognitive symptoms after apparent recovery from acute CO poisoning. DNS is characterized by various symptoms and signs, including mental deterioration, cognitive dysfunction, amnesia, gait disturbance, mutism, urinary or fecal incontinence, psychosis, depression, and Parkinsonism (22).

### 2.4. Study outcomes

The primary outcome was to assess the value of quantitative pupillary reactivity (NPi and qPLR) compared to that of sPLR in predicting neurocognitive outcome 1 month after acute CO poisoning.

### 2.5. Statistical analysis

Continuous variables were expressed as medians (interquartile range, IQR) or mean with standard deviation using the Mann–Whitney U test, Kruskal-Wallis test, or t test, if appropriate. Categorical variables were expressed as frequency and percentage and analyzed using the Chi-square or Fisher’s exact tests. The serial measurements of pupillometry parameters were compared between patients with favorable outcomes and those with poor neurocognitive outcomes. A receiver operating characteristic (ROC) curve analysis was performed to evaluate the performance of NPi, qPLR, and sPLR in predicting poor outcomes. *p*-values <0.05 were considered statistically significant. All statistical analyses were conducted using SPSS for Windows version 25.0 (IBM SPSS inc., Chicago, IL, United States) and R studio and R version 4.0.3, and the sample size was calculated using PASS version 14.

## 3. Results

### 3.1. Characteristics of the study population

This study included 104 of 230 patients with acute CO poisoning who visited the ED between August 2019 and December 2020 (Figure 1). Table 1 shows the baseline characteristics of the patients. Among the included patients, 83 (79.8%) had favorable neurocognitive outcomes. Patients with favorable outcomes were significantly younger (mean, 43.7 vs. 57.7 years, *p* < 0.001), were more likely to have a non-fire CO poisoning (100% vs. 85.7%, *p* = 0.007), and had a significantly shorter duration of CO exposure (median, 4.3 h vs. 10.5 h, *p* < 0.001) than those with poor outcomes. Comorbid conditions did not differ between those with favorable and poor outcomes. However, shock (3.6% vs. 28.6%, *p* < 0.001) and intubation (27.7% vs. 76.2%, *p* < 0.001) occurred less frequently in patients with favorable outcomes than in those with poor outcomes.

**Table 1:** Baseline characteristics of the included patients in each group.

Variables	Total (*n* = 104)	Favorable outcome (*n* = 83, 79.8%)	Poor outcome (*n* = 21, 20.2%)	*P*-value
Age (year)	46.5 ± 16.8	43.7 ± 15.8	57.7 ± 16.5	<0.001
Female sex (%)	37 (35.6)	28 (33.7)	9 (42.9)	0.435
Suicide attempt (%)	67 (64.4)	53 (63.9)	14 (66.7)	0.810
Source of carbon monoxide (%)				0.007
Fire	3 (2.9)	0 (0.0)	3 (14.3)	
Non-fire	101 (97.1)	83 (100.0)	18 (85.7)	
Duration of exposure to carbon monoxide (h)	5.4 (1.9–8.0)	4.3 (1.5–8.0)	10.5 (8.0–13.5)	<0.001
Drugs use during admission				
Sedative drug	26 (25.0)	13 (15.7)	13 (61.9)	<0.001
Midazolam (%)	14 (13.5)	4 (4.8)	10 (47.6)	<0.001
Midazolam dose (mg)	375 (250–450)	325 (127–425)	375 (250–500)	0.477
Lorazepam (%)	25 (24.0)	12 (14.5)	13 (61.9)	<0.001
Lorazepam dose (mg)	20 (16–28)	18 (14–26)	20 (16–36)	0.582
Dexmedetomidine (%)	23 (22.1)	11 (13.3)	12 (57.1)	<0.001
Dexmedetomidine dose (mcg)	200 (200–500)	300 (200–500)	200 (200–700)	0.947
NM blockers	13 (12.5)	5 (6.0)	8 (38.1)	<0.001
Cisatracurium (%)	13 (12.5)	5 (6.0)	8 (38.1)	<0.001
Cisatracurium dose (mg)	120 (105–210)	105 (105–110)	205 (113–213)	0.238
Rocuronium (%)	1 (1.0)	0 (0.0)	1 (1.0)	0.202
Rocuronium dose (mg)	10	-	10	-
Opioid	3 (2.9)	2 (2.4)	1 (4.8)	0.495
Fentanyl (%)	3 (2.9)	2 (2.4)	1 (4.8)	0.495
Fentanyl dose (mcg)	50 (50–150)	100 (50–150)	50	0.480
Remifentanil (%)	1 (1.0)	0 (0.0)	1 (1.0)	0.202
Remifentanil dose (mcg)	28	-	28	-
Coexisting conditions (%)				
Diabetes mellitus	16 (15.4)	13 (15.7)	33 (14.3)	0.876
Hypertension	20 (19.2)	14 (16.9)	6 (28.6)	0.224
Chronic kidney disease	0 (0.0)	0 (0.0)	0 (0.0)	-
Heart disease	3 (2.9)	2 (2.4)	1 (4.8)	0.565
Advanced liver disease	2 (1.9)	1 (1.2)	1 (4.8)	0.365
Psychiatric disease	24 (23.1)	22 (26.5)	2 (9.5)	0.099
Initial symptoms and patient status (%)				
Loss of consciousness	86 (82.7)	66 (79.5)	20 (95.2)	0.089
Shock (%)	9 (8.7)	3 (3.6)	6 (28.6)	<0.001
Intubation (%)	39 (37.5)	23 (27.7)	16 (76.2)	<0.001
GCS	12 (8–15)	13 (10–15)	8 (5–10)	<0.001
Time from rescue to 1^st^ HBO_2_ (h)	4.2 (2.9–6.0)	4.2 (2.8–6.0)	4.5 (3.3–17.0)	0.281
HBO_2_ number within 24 h after ED arrival	2.0 (1.5–2.0)	2.0 (2.0–2.0)	2.0 (1.0–2.0)	0.069
HBO_2_ number during admission	4 (2–6)	4 (2–6)	3 (2–6)	0.871
Laboratory tests				
Initial carboxyhemoglobin (%)	8.0 (3.4–23.5)	7.9 (3.5–23.5)	10.0 (2.1–30.2)	0.789
Troponin I (ng/mL)	0.005 (0.000–0.528)	0.003 (0.000–0.238)	0.564 (0.003–2.227)	0.037
Bicarbonate (mEq/L)	21.4 (18.5–23.6)	22.4 (19.2–24.0)	18.1 (15.6–20.6)	0.001
Lactate (mg/dL)	2.5 (1.6–3.8)	2.3 (1.5–3.6)	2.7 (2.2–5.6)	0.088
Complications				
Rhabdomyolysis	28 (26.9)	12 (14.5)	16 (76.2)	<0.001
Acute kidney injury	9 (8.7)	4 (4.8)	5 (23.8)	0.006
Pneumonia	20 (19.2)	9 (10.8)	11 (52.4)	<0.001
GDS				
At 1 month	1 (1–3)	1 (1–2)	6 (5–7)	<0.001
^a^At 6 months	1 (1–3)	1 (1–1)	5 (4–7)	<0.001

Data are expressed as frequency (percentage), mean ± standard deviation, and median (interquartile range). CO, carbon monoxide; NM, neuromuscular; GCS, Glasgow coma scale; HBO_2_, hyperbaric oxygen therapy; ED, emergency department; GDS, Global Deterioration Scale.

a GDS score at 6 months was measured for 72 patients.

Compared to patients with favorable outcomes, those with poor outcomes had significantly lower GCS scores (median, 13 vs. 8, *p* < 0.001), and were treated with more sedatives (15.7% vs. 61.9%, *p* < 0.001) or neuromuscular blockers (6.0% vs. 38.1%, *p* < 0.001). However, the average dose of the drugs did not differ between the patients with favorable and poor outcomes. In addition, the patients with poor outcomes had significantly higher serum troponin I (median, 0.003 vs. 0.564, *p* = 0.037) and lower serum bicarbonate (median, 22.4 vs. 18.1, *p* = 0.001) levels. All investigated complications, such as pneumonia, acute kidney injury, or rhabdomyolysis, were also more frequent in patients with poor outcomes.

### 3.2. Characteristics of the quantitative pupillary variables

Pupillometry data were compared between the two groups (Table 2). The lowest NPi values within 24 h after admission, or the lowest one during the admission periods did not differ between patients with favorable outcomes and those with poor outcomes (mean, 3.91 vs. 3.58, *p* = 0.221 and 3.88 vs. 3.47, *p* = 0.132, respectively). However, the lowest qPLR within 24 h after admission and the lowest one over the admission periods were significantly higher in patients with favorable outcomes than in those with poor outcomes (mean, 21.2% vs. 15.0%, *p* = 0.006 and 21.0% vs. 14.8%, *p* = 0.006). sPLR was not significantly different between the two groups (*p* = 0.077). Interestingly, two patients with non-reactive sPLR had favorable outcomes. In patient 1, sPLR was assessed as non-reactive on admission. However, NPi values were 2.5 (upon admission) and 4.1 (at 24 h); qPLR values were 18% (upon admission) and 26% (at 72 h). In patient 2, sPLR was non-reactive up to 6 h, became sluggish at 12 h, and improved to reactive subsequently. NPi and qPLR values were 1.5 and 15% (upon admission) and improved to more than 2.1 and 20% (at 48 h), respectively. When analyzed at each point in time, qPLR values measured at 12 and 24 h were higher in patients with favorable outcomes than in those with poor outcomes (mean, 32.2 ± 9.7 vs. 23.4 ± 11.0, *p* < 0.001; 28.8 ± 9.7 vs. 21.8 ± 13.3, *p* = 0.011, respectively; Table 3). Other pupillometer variables, including maximum pupil size, minimum pupil size, and latency, were compared between the two groups. The representative values at 24 h or during the whole monitoring time did not differ between patients with favorable outcomes and those with poor outcomes. However, the patients with poor outcomes had a significantly lower constriction velocity and worse maximum constriction velocity during the whole monitoring time compared to those with favorable outcomes (median, 0.79 vs. 1.49, *p* = 0.001; 1.13 vs. 2.12. *p* = 0.002, respectively). In addition, they had lower dilatation velocity at 24 h or during the whole monitoring time (median, 0.72 vs. 0.94, *p* = 0.032; 0.33 vs. 0.63. *p* = 0.010, respectively; Supplementary Table S2).

**Table 2:** Pupillometer data recorded at initial and serial evaluations after carbon monoxide poisoning categorized according to outcome groups.

Variables	Favorable outcome (*n* = 83, 79.8%)	Poor outcome (*n* = 21, 20.2%)	*P*-value
NPi			
24-h lowest values	3.91 ± 0.71	3.58 ± 1.16	0.221
Total lowest values	3.88 ± 0.73	3.47 ± 1.15	0.132
qPLR (%)			
24-h lowest values	21.2 ± 8.9	15.0 ± 9.5	0.006
Total lowest values	21.0 ± 8.7	14.8 ± 9.7	0.006
Standard PLR (%)			
24-h lowest values			0.077
Reactive	81 (97.6)	19 (90.5)	
Sluggish	0 (0.0)	2 (9.5)	
Non-reactive	2 (2.4)	0 (0.0)	
Total lowest values			0.077
Reactive	81 (97.6)	19 (90.5)	
Sluggish	0 (0.0)	2 (9.5)	
Non-reactive	2 (2.4)	0 (0.0)	

NPi, neurological pupil index; qPLR, quantitative pupillary light reflex.

**Table 3:** Pupillometer data recorded at each point in time after carbon monoxide poisoning categorized according to outcome groups.

Variables	Favorable outcome (*n* = 83, 79.8%)	Poor outcome (*n* = 21, 20.2%)	*P*-value
NPi			
0 h (*n* = 104)	4.17 ± 0.52	3.81 ± 1.12	0.163
6 h (*n* = 96)	4.21 ± 0.67	4.10 ± 1.02	0.639
12 h (*n* = 94)	4.29 ± 0.74	4.11 ± 1.10	0.487
24 h (*n* = 87)	4.28 ± 0.59	4.14 ± 0.91	0.516
48 h (*n* = 62)	4.18 ± 0.76	3.95 ± 1.05	0.342
72 h (*n* = 41)	4.26 ± 0.69	4.02 ± 1.18	0.538
qPLR (%)			
0 h (*n* = 104)	26.6 ± 8.4	25.0 ± 8.6	0.420
6 h (*n* = 96)	28.2 ± 9.4	25.0 ± 11.3	0.194
12 h (*n* = 93)	32.2 ± 9.7	23.4 ± 11.0	<0.001
24 h (*n* = 87)	28.8 ± 9.7	21.8 ± 13.3	0.011
48 h (*n* = 62)	32.5 ± 9.7	26.3 ± 14.5	0.105
72 h (*n* = 41)	29.3 ± 9.7	26.8 ± 12.9	0.511

NPi, neurological pupil index; qPLR, quantitative pupillary light reflex.

**Figure 2:**
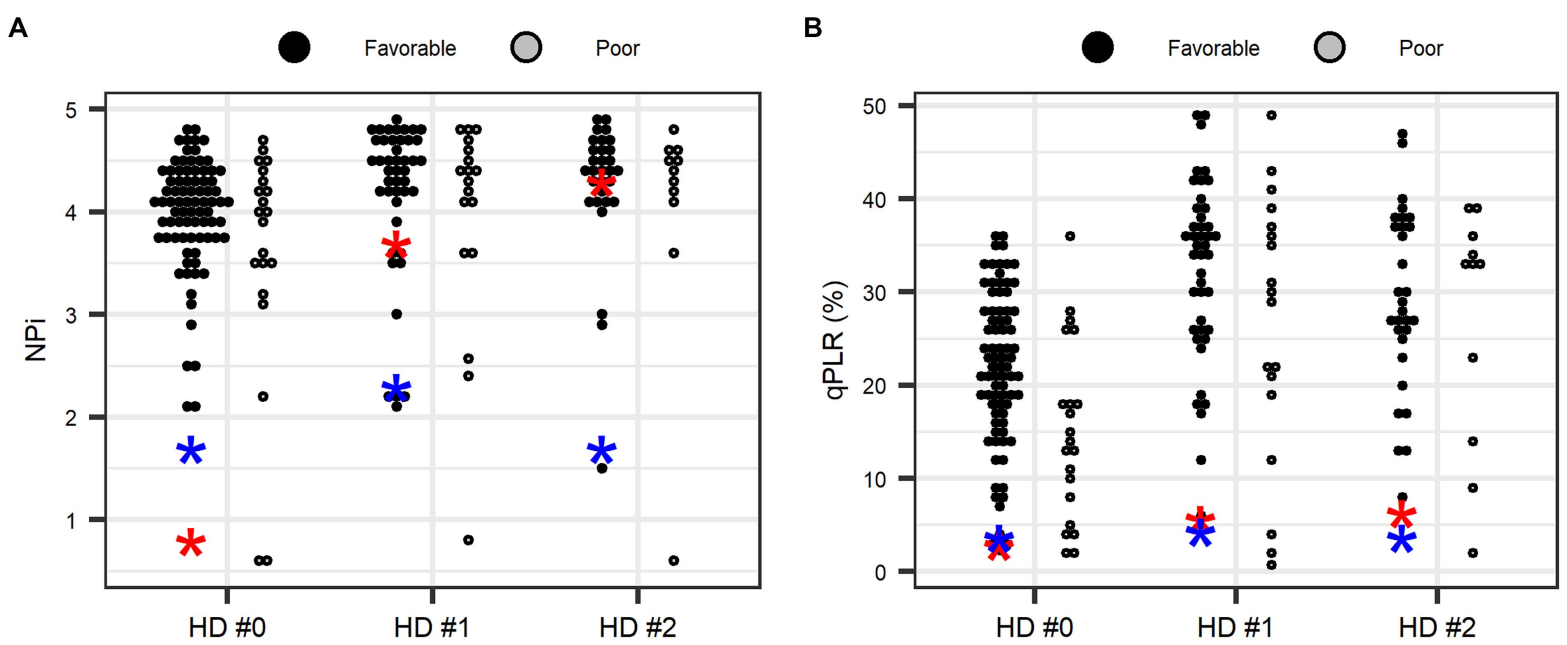
Distributions of neurological pupil index **(A)** and quantitative pupillary light reflex **(B)** during the first 3 days after carbon monoxide poisoning according to 1-month patient outcomes. NPi, neurological pupil index; qPLR, quantitative pupillary light reflex. *Blue and red colors indicate two patients with non-reactivity in standard PLR. Values represent the lowest value in each day.

ROC analysis was performed to compare pupillometer data in predicting neurocognitive outcome at 1 month (Table 4). The AUC value was 0.70 for qPLR [(95% confidence interval (CI): 0.60–0.78)], 0.54 for NPi (95% CI: 0.44–0.64) and 0.51 for sPLR [0.51 (95% CI: 0.41–0.61)]. qPLR was significantly superior to sPLR in predicting poor neurocognitive outcomes (GDS 5–7) at 1 month after CO poisoning (*p* = 0.007). However, qPLR was not superior to NPi in predicting a poor prognosis (*p* = 0.126). A cut-off of NPi < 1 yielded a negative predictive value of 81.2% (95% CI: 72.2–88.3) and a positive predictive value of 66.7% (95% CI: 9.4–99.2), with a specificity of 98.8% (95% CI: 93.5–100) and a sensitivity of 9.5% (95% CI: 1.2–30.4) for the prediction of poor outcomes. The qPLR <18% yielded a high negative predictive value [91.8% (95% CI: 81.9–97.3)] with a sensitivity of 76.2% (95% CI: 52.8–91.8) and a specificity of 67.5% (95% CI 56.3–77.4) in predicting poor outcomes. The specificity of bilaterally absent sPLR (only 2 of 104 patients) for poor outcome was 97.6% at the ED, with sensitivity of 0% and positive predictive value of 0%. Figure 2 illustrates the distributions of individual NPi and qPLR from day 1 to 3 after CO poisoning in both outcome groups. Furthermore, the results of NPi and qPLR were further analyzed in the context of mental status using GCS. GCS was dichotomized based on its median value of 12. Among the patients with decreased mental status (GCS ≤ 12), the AUC values of NPi < 1 and qPLR <18% improved [0.75 (95% CI: 0.66–0.83) and 0.80 (95% CI: 0.71–0.87), respectively] (Table 4).

**Table 4:** Prediction of poor neurocognitive outcome at 1 month.

Variables	Cut-off value	Specificity % (95% CI)	Sensitivity % (95% CI)	NPV % (95% CI)	PPV % (95% CI)	False-positive rate % (95% CI)	AUC (95% CI)
Only pupillometry parameters							
NPi							
24-h lowest values	3.6	79.5 (69.2–87.6)	42.9 (21.8–66.0)	84.0 (73.7–91.5)	31.0 (15.3–50.8)	69.0 (49.2–84.7)	0.57 (0.47–0.66)
	1	98.8 (93.5–100.0)	9.5 (1.2–30.4)	81.2 (72.2–88.3)	66.7 (9.4–99.2)	33.3 (0.8–90.6)	0.54 (0.44–0.64)
Total lowest values	3.6	78.3 (67.9–86.6)	47.6 (25.7–70.2)	84.9 (74.6–92.2)	32.3 (16.7–51.4)	67.7 (48.6–83.3)	0.60 (0.50–0.70)
	1	98.8 (93.5–100.0)	9.5 (1.2–30.4)	81.2 (72.2–88.3)	66.7 (9.4–99.2)	33.3 (0.8–90.6)	0.54 (0.44–0.64)
qPLR %							
24-h lowest values	18	67.5 (56.3–77.4)	76.2 (52.8–91.8)	91.8 (81.9–97.3)	37.2 (23.0–53.3)	62.8 (46.7–77.0)	0.70 (0.60–0.78)
Total lowest values	18	67.5 (56.3–77.4)	76.2 (52.8–91.8)	91.8 (81.9–97.3)	37.2 (23.0–53.3)	62.8 (46.7–77.0)	0.70 (0.60–0.78)
sPLR*							
24-h lowest values/ Total lowest values		97.6 (91.6–99.7)	0.0 (0.0–16.1)	79.4 (70.3–86.8)	0.0 (0.0–84.2)	100.0 (15.8–100.0)	0.51 (0.41–0.61)
Pupillometry parameters combined with GCS ≤ 12							
NPi							
24-h lowest values	3.6	50.6 (39.4–61.8)	90.5 (69.6–98.8)	95.5 (84.5–99.4)	31.7 (20.3–45.0)	68.3 (55.0–79.7)	0.74 (0.65–0.82)
	1	50.6 (39.4–61.8)	90.5 (69.6–98.8)	95.5 (84.5–99.4)	31.7 (20.3–45.0)	68.3 (55.0–79.7)	0.72 (0.63–0.81)
Total lowest values	3.6	50.6 (39.4–61.8)	90.5 (69.6–98.8)	95.5 (84.5–99.4)	31.7 (20.3–45.0)	68.3 (55.0–79.7)	0.75 (0.66–0.83)
	1	50.6 (39.4–61.8)	90.5 (69.6–98.8)	95.5 (84.5–99.4)	31.7 (20.3–45.0)	68.3 (55.0–79.7)	0.72 (0.63–0.81)
qPLR %							
24-h lowest values	18	79.5 (69.2–87.6)	66.7 (43.0–85.4)	90.4 (81.2–96.1)	45.2 (27.3–64.0)	54.8 (36.0–72.7)	0.80 (0.71–0.87)
Total lowest values	18	79.5 (69.2–87.6)	66.7 (43.0–85.4)	90.4 (81.2–96.1)	45.2 (27.3–64.0)	54.8 (36.0–72.7)	0.80 (0.71–0.87)
sPLR*							
24-h lowest values/ Total lowest values		53.0 (41.7–64.1)	90.5 (69.6–98.8)	95.7 (85.2–99.5)	32.8 (21.0–46.3)	67.2 (53.7–79.0)	0.72 (0.62–0.80)

CI, confidence interval; NPV, negative predictive value; PPV, positive predictive value; AUC, area under curve; NPi, neurological pupil index; qPLR, quantitative pupil light reflex; sPLR, standard pupil light reflex; GCS, Glasgow coma scale.

* Positive means non-reactive.

The individual serial pupillometry values and brain diffusion-weighted images (DWI; taken in the ED) of three patients with NPi < 1 are illustrated in the Supplementary Figures S1, S2. Two of the three patients with NPi < 1 had poor outcomes and showed diffuse bilateral lesions on brain DWI, whereas one patient with a favorable outcome had globus pallidus and non-diffuse lesions.

We compared the pupillometry data among the patients without sequelae (favorable outcome), those with DNS, and those with permanent neurologic sequelae (PNS; Supplementary Table S3 and Supplementary Figure S3). The NPi did not differ among the three groups. However, the lowest qPLR at 24-h (13.0, *p* = 0.038) or during the whole monitoring period (13.0, *p* = 0.041) were lower in the PNS group. In the *post hoc* test, the median qPLR was significantly lower in patients with PNS compared to those without sequelae group (13.0 vs. 21.0, *p* = 0.033 at 24 h; 13.0 vs. 21.0, *p* = 0.034 for total monitoring time, respectively). Constriction velocity and maximum dilatation velocity were the highest in patients without neurological sequelae, whereas the size of pupil or latencies did not differ among three groups (Supplementary Table S4).

## 4. Discussion

In this study, we showed that NPi and qPLR were superior to sPLR in predicting poor neurological outcomes 1 month after CO poisoning. The cut-off value of pupillometer variables for predicting poor neurological outcome was NPi of 1.0 or qPLR of 18%. Moreover, the power of those variables was more robust in patients with a decrease in mental status (GCS ≤12).

A previous report showed that a lack of sPLR was associated with poor neurological outcomes 30 days after CO poisoning (4). However, as described above, The use of sedatives may have affected the PLR, but NM blockers do not (14). However, we used the NPi values and qPLR to correlate the neurocognitive outcomes because these values were relatively less affected by the use of drugs compared to sPLR. This suggests that NPi and qPLR measurements taken using a pupillometer may be more accurate in assessing pupillary reactivity.

Neurocognitive sequelae are the most important neurological complication after CO poisoning, and its incidence is estimated as high as 50% among the survivors after acute CO poisoning (23). A previous report showed that a loss of sPLR was a predictor for poor neurocognitive outcomes (4). In line with this, our results also showed that pupillometer variables (NPi and qPLR) were more useful in identifying patients with poor functional outcomes. Indeed, two patients in our study had bilateral fixed pupils on sPLR assessment upon admission, which was regarded as reactive on the pupillometer. Moreover, qPLR had a higher AUC than sPLR, suggesting that it was more powerful than sPLR in identifying patients with poor outcomes. In the results, more patients with poor functional outcomes were treated with sedatives or neuromuscular blockers. Given that NPi is less affected by the sedatives compared to qPLR, the difference of qPLR between the patients with and without favorable outcomes might be due to the concomitant use of sedatives. This should be interpreted in caution.

In this study, the lowest NPi value did not differ between patients with favorable outcomes and those with poor outcomes on HD 0. However, the NPi values improved to ≥1.5 on HD 1 and HD 2 in patients with favorable outcomes. Therefore, using a cut-off point of NPi of 1.0 during 72 h, the specificity of NPi for predicting poor outcomes was 98.8%, with a positive predictive value of 66.7%. Opioids decrease the size of the pupil and may alter the qPLR partly mediated by small pupil size (24). The NPi, however, is not affected by pupil size alterations. Therefore, NPi would be more useful for accurate prognosis in conditions with concomitant use of sedatives including opioids.

The cut-off value of NPi of 1.0 was lower than that in a previous cardiac arrest study, which showed an NPi of <2.0 at any time between HD 0 and 2 following hospital admission, with 100% specificity for the prediction of unfavorable neurological outcomes at 3 months (15). In studies by Kim et al. (25) and Jeon et al. (26), the presence of acute brain lesions on brain DWI was significantly associated with poor neurocognitive outcomes. In our study, two of three patients with an NPi < 1 had diffuse bilateral cortico-subcortical lesions on brain DWI and showed a poor prognosis. Therefore, diffuse brain DWI lesions on top of an NPi value of <1 may suggest a poor prognosis in patients with CO poisoning.

There are several hypotheses as to why neither NPi nor qPLR had a high prediction power in CO poisoning compared that in *CA*. First, the degree of injury is usually less severe and sometimes reversible in CO poisoning (hypoxic) compared to that in CA (anoxic). HBO_2_ often demonstrates a reversible effect on inflammation or mitochondrial dysfunction after CO poisoning, which may have affected the results in this study (27). Second, the reticular activating system in the upper brainstem is relatively resistant to hypoxic damage compared to the cerebral cortex (28). Therefore, abnormal PLR can only be identified in patients with severe neurological injuries and with significant damage in the cerebral cortex (29). Given that the degree of injury is more severe in patients with CA, it is plausible that the percentage of patients with brainstem dysfunction on top of cortical damage is higher in CA than in CO poisoning.

There are several limitations in this study. First, this was a single-centered study with relatively small numbers of patients. Second, the length of hospital stay was not identical in all patients. Therefore, a serial pupillometer exam was not performed in all patients up to 72 h. Third, given the limitations of the observational study, there may be some confounders that we did not adjust for in the analysis. Fourth, Sedatives and neuromuscular blockers were used to support mechanical ventilation. Therefore, patients with more severe injuries were more likely to be on those medications, which may have affected the pupillary reactivity. Nevertheless, this is the first study to evaluate the role of a pupillometer in predicting neurocognitive outcomes among the prospectively collected consecutive patients with CO poisoning.

NPi and qPLR was superior to sPLR in predicting poor neurocognitive outcomes after CO poisoning. The power of Low NPi <1.0 or low qPLR <18% became more robust in patients with a decrease in mental status, with a low false-positive rate and a high specificity.

## Data availability statement

The original contributions presented in the study are included in the article/Supplementary material, further inquiries can be directed to the corresponding author.

## Ethics statement

The study protocol was approved by the institutional review board of Wonju Severance Christian Hospital (approval number: CR319039). The patients/participants provided their written informed consent to participate in this study.

## Author contributions

YC and S-BK conceived the study, designed the trial, drafted the manuscript, took responsibility for the paper as a whole, and supervised the conduct of the trial and data collection. YC and DL obtained research funding. YC undertook recruitment of participating centers and patients and managed the data, including quality control. T-HG provided statistical advice on study design and analyzed the data. All authors contributed substantially to its revision.

## Funding

This work was supported by the Seoul National University Bundang Hospital Research Fund [grant no. 14–2018-029] and National Research Foundation of Korea, which was funded by the Korean government Ministry of Science and Information and Communications Technology [grant no. NRF-2021R1A2C200492211].

## Conflict of interest

The authors declare that the research was conducted in the absence of any commercial or financial relationships that could be construed as a potential conflict of interest.

## Publisher’s note

All claims expressed in this article are solely those of the authors and do not necessarily represent those of their affiliated organizations, or those of the publisher, the editors and the reviewers. Any product that may be evaluated in this article, or claim that may be made by its manufacturer, is not guaranteed or endorsed by the publisher.
