# Supplementary material for: Quantitative pupillary light reflex assessment for prognosis of carbon monoxide poisoning
Source: Front Med (Lausanne). 2023 Feb 28;10:1105705. doi: 10.3389/fmed.2023.1105705 (PMC10011084; doi:10.3389/fmed.2023.1105705)
Supplement: Supplementary file 1 [file Data_Sheet_1.pdf]

Supplementary Material

Quantitative Pupillary Light Reflex Assessment for Prognosis of Carbon Monoxide Poisoning

Yong Sung Cha[1,2*], Sang-Bae Ko[3*], Tae-Hwa Go[4], Dong Keon Lee[5]

*** Correspondence:** Yong Sung Cha:emyscha@yonsei.ac.kr; Sang-Bae Ko: sangbai1378@gmail.com

Supplementary Method 1. Variable Definitions.

Shock was diagnosed when a vasopressor was needed to resuscitate the patient and lactate levels exceeded 2.0 mmol/L. The information on the duration of carbon monoxide (CO) exposure obtained from patients' guardians was the estimated maximum duration of CO exposure, measured from the time of normal consciousness to the time of rescue. The CO exposure-to-hyperbaric oxygen therapy (HBO_2) interval was defined as the interval from the time of rescue from the CO source to the start of the first HBO_2 session.

Supplementary Method 2. Global Deterioration Scale.

The Global Deterioration Scale (GDS) is a validated reliable instrument used for the description of the clinical progression of dementia.[1] It is also used to determine the prognoses of patients with CO poisoning[2] and those of patients with severe chronic obstructive pulmonary disease, Alzheimer's disease, and vasculopathy-related dementia.[1,3-5]

Although the GDS score is not as diverse as a CO battery, it has the advantage of allowing the detection of neurocognitive functions, such as memory and concentration, as well as activities of daily living, through interviews. Moreover, undergoing many neurocognitive function tests may be difficult for patients with sequelae. The Short-Form General Health Survey-36, a commonly used testing tool, has a set of self-report questions; however, it is limited in the evaluation of patients with severe neurological impairments as it requires the ability to understand and address the questions. Digit span, trail making, and clock drawing are good evaluation tools; however, they require short-term memory and visuospatial functions. Therefore, the GDS score can be used for all patients with CO poisoning, regardless of the severity of the poisoning. The scale consists of seven scores, with higher scores indicating greater severity.

References

1. Reisberg B, Ferris SH, de Leon MJ, et al: The Global Deterioration Scale for assessment of primary degenerative dementia. *Am J Psychiatry* 1982; 139:1136-1139

2. Kim SJ, Thom SR, Kim H, et al: Effects of adjunctive therapeutic hypothermia combined with hyperbaric oxygen therapy in acute severe carbon monoxide poisoning. *Crit Care Med* 2020; 48:e706-e714

3. Paul RH, Cohen RA, Moser DJ, et al: The global deterioration scale: Relationships to neuropsychological performance and activities of daily living in patients with vascular dementia. *J Geriatr Psychiatry Neurol* 2002; 15:50-54

4. Eisdorfer C, Cohen D, Paveza GJ, et al: An empirical evaluation of the Global Deterioration Scale for staging Alzheimer's disease. *Am J Psychiatry* 1992; 149:190-194

5. Ozge C, Ozge A, Unal O: Cognitive and functional deterioration in patients with severe COPD. *Behav Neurol* 2006; 17:121-130

(A) Patient 1

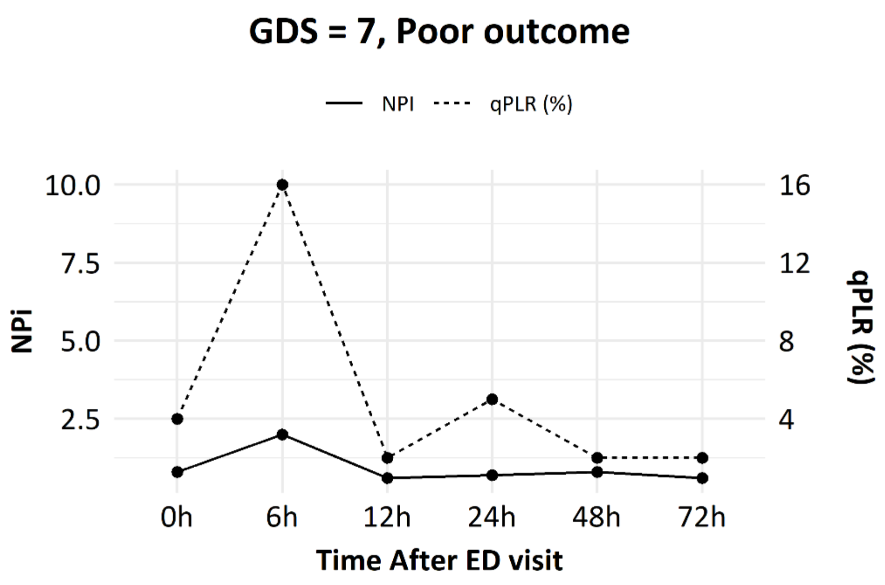

(B) Patient 2

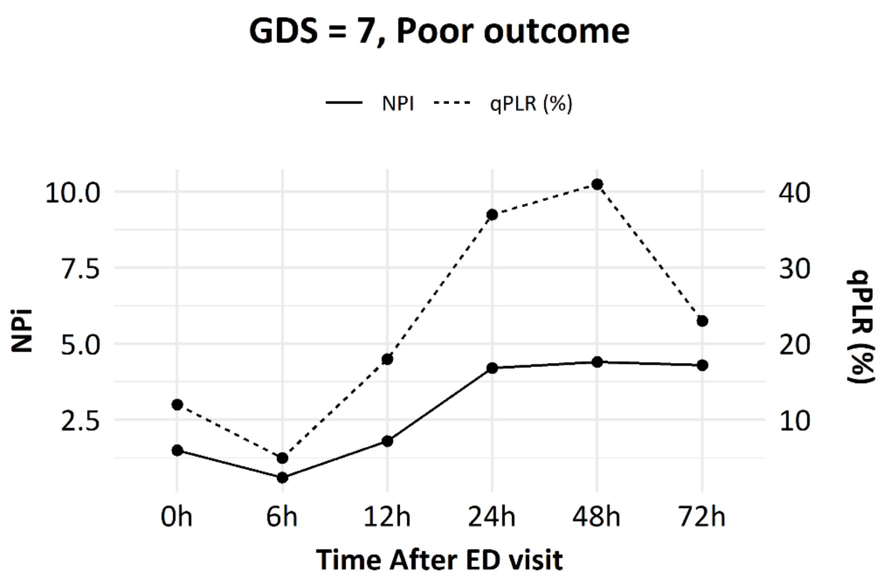

(C) Patient 3

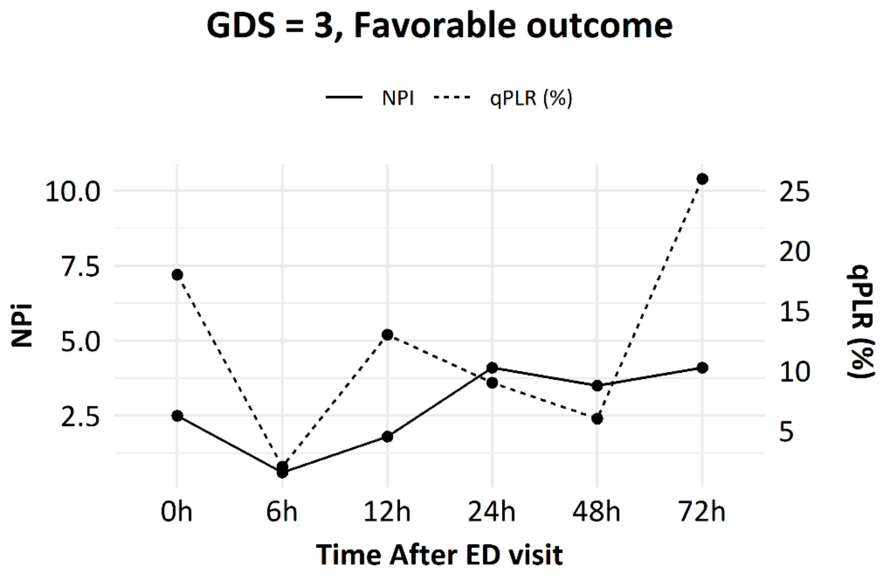

NPi = Neurological Pupil index, GDS = Global Deterioration Scale, ED = emergency department.

Supplementary Figure 1. Individual serial neurological pupil index and quantitative pupillary light reflex of patients with neurological pupil index < 1**.**

(A) Patient 1

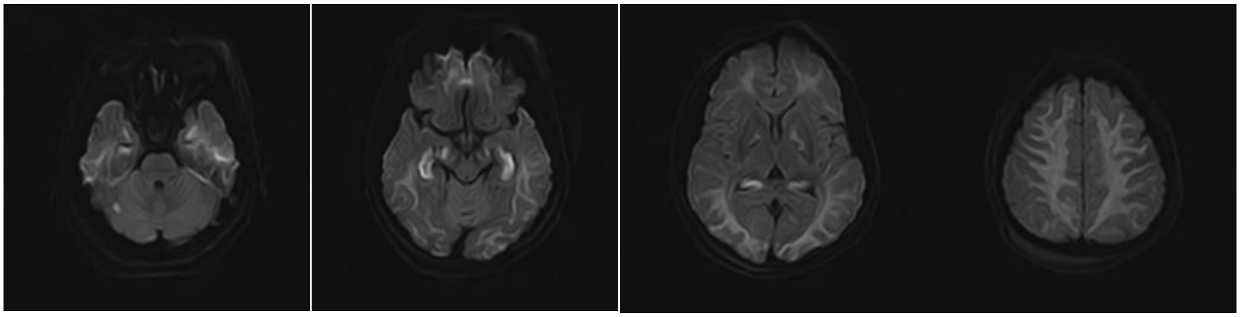

(B) Patient 2

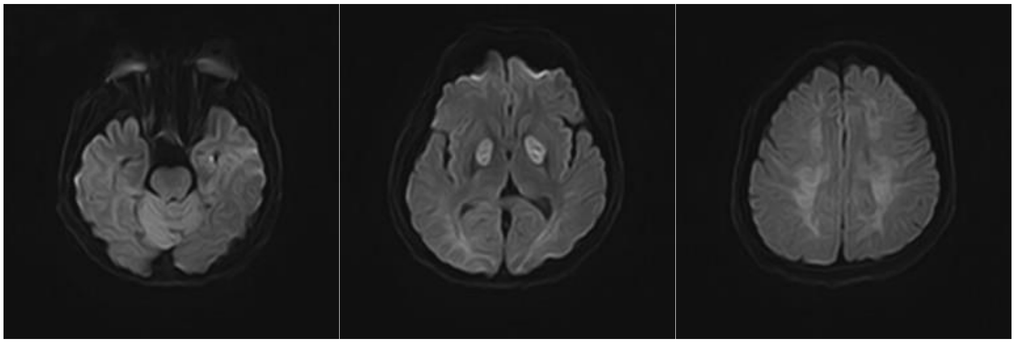

(C) Patient 3

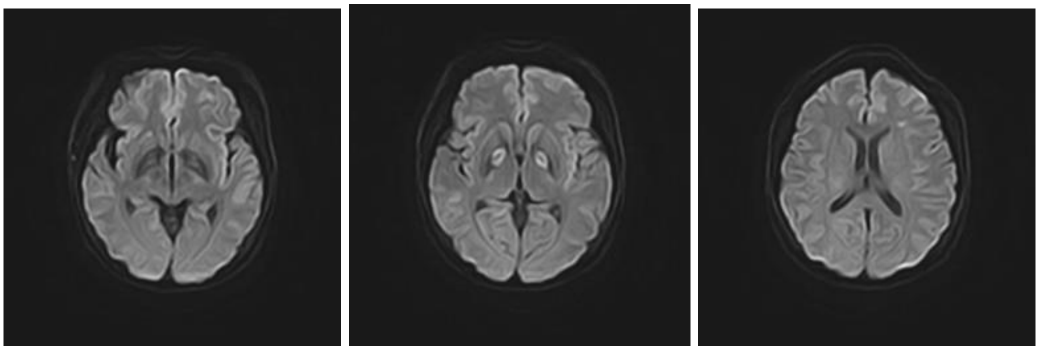

Supplementary Figure 2. Individual brain diffusion-weighted image of three patients with neurological pupil index < 1. NPi = Neurological Pupil index.

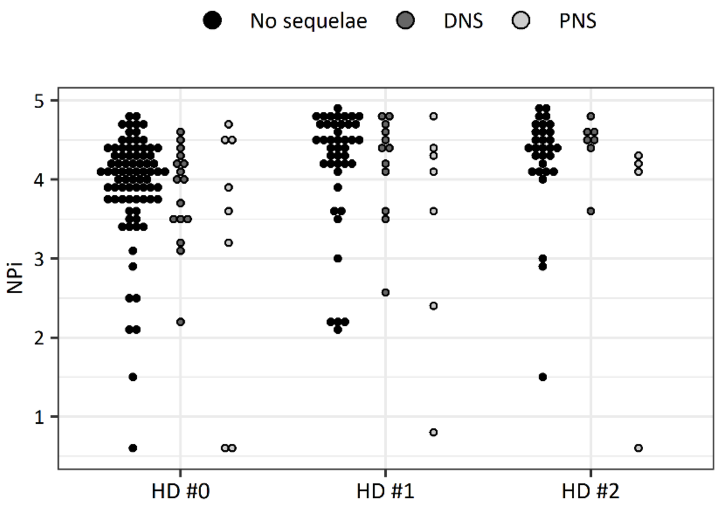

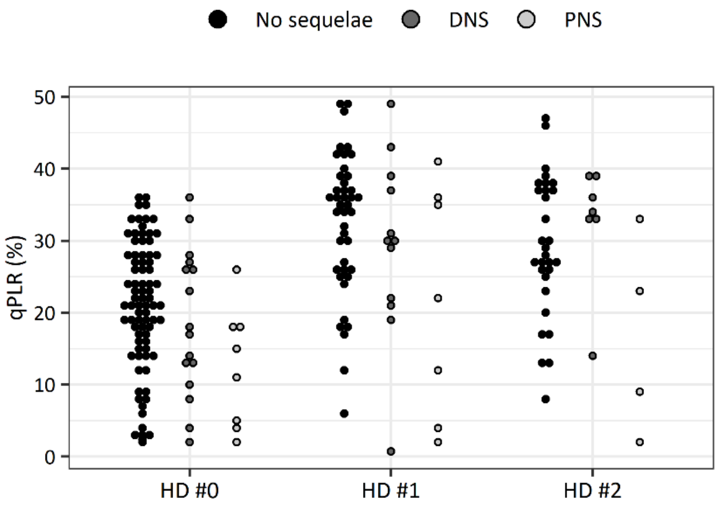

Supplementary Figure 3. Distribution of neurological pupil index according to 1-month patient outcome.

NPi = Neurological Pupil index, DNS = delayed neurocognitive sequelae, PNS = permanent neurologic sequalae

Supplementary Table 1. Global Deterioration Scale.

Stage	Cognitive dysfunction	Clinical characteristics
1	No cognitive decline	*Patients appear clinically normal*<br><br>No complaints of memory deficits.<br><br>No evident memory deficit on clinical interview.
2	Very mild cognitive decline	*Patients complain of memory deficits*<br><br>Most frequently, patients:<br><br>(a) forget where they have placed familiar objects<br><br>(b) forget the name of someone they formerly knew well.<br><br>No objective evidence of memory deficit on clinical interview.<br><br>No objective deficits in employment or social situations.<br><br>Patients display appropriate concern about their symptoms.
3	Mild cognitive decline	*Earliest clear-cut deficits*<br><br>Objective evidence of memory deficit was obtained only through an intensive interview conducted by a trained geriatric psychiatrist. Concentration deficit may be evident on clinical testing.<br><br>Patients may demonstrate a reduced ability to:<br><br>(a) remember names upon introduction to new people<br><br>(b) retain information after reading a passage from a book<br><br>Decreased performance becomes manifest in demanding employment and social situations. Examples may include:<br><br>(a) coworkers becoming aware of the patient's relatively poor performance<br><br>(b) difficulties in finding words and names becoming evident to intimate acquaintances<br><br>(c) losing or misplacing objects of value

		(d) getting lost when traveling to unfamiliar locations<br><br>The subtlety of the clinical symptoms may be exacerbated by denial that often manifests in these patients. Mild to moderate anxiety also accompany the symptoms, typically when patients are forced to cope with challenging employment and social demands that they find they can no longer negotiate.
4	Moderate cognitive decline	*<u>Clear-cut deficits on careful clinical interview</u>*<br><br>Deficits manifest in many areas, such as:<br><br>(a) concentration deficit elicited in serial subtractions<br><br>(b) decreased knowledge of current events and recent life events<br><br>(c) upon careful questioning, patients may exhibit a deficit in the memory of their personal history<br><br>(d) decreased ability to travel alone and manage finances<br><br>Patients can no longer perform complex tasks accurately and efficiently. However, certain abilities remain preserved, such as:<br><br>(a) orientation to time and people<br><br>(b) familiar persons and faces can be distinguished from strangers<br><br>(c) ability to travel to familiar locations<br><br>Denial is often the dominant defense mechanism. The evident decline in patients' intellectual and cognitive capacities is overwhelming for full conscious acceptance and recognition. A flattening of effect and withdrawal from previously challenging situations are observed.
5	Moderately severe cognitive decline	*<u>Patients can no longer survive without some assistance</u>*<br><br>During interviews, patients are unable to recall a major relevant aspect of their current lives. Examples include:<br><br>(a) difficulty recalling their address or telephone number, names of close family members, such as grandchildren, or the

		name of the high school or university from which they graduated<br><br>(b) some disorientation with time (date, day of the week, season) or location<br><br>(c) well-educated patients may have difficulty counting backwards from 40 by fours or from 20 by twos.<br><br>Patients retain the knowledge of many major facts regarding themselves and others. For example, they invariably know their own names and generally know their spouse and children's names. They require no assistance with toileting and eating, but may have difficulty choosing the proper clothing to wear and may occasionally clothe themselves improperly (e.g., putting their shoes on the wrong feet).
6	Severe cognitive decline	*Patients may occasionally forget the name of their spouse, on whom they depend entirely for survival*<br><br>Patients are largely unaware of all recent events and experiences in their lives.<br><br>They retain some knowledge of their past, although this knowledge is uncertain. They are generally unaware of their surroundings, the year, or the season, and may have difficulty counting backwards and sometimes forward from 10. Thus, patients require substantial assistance with activities of daily living. These symptoms are quite variable and include:<br><br>(a) delusional behavior (e.g., patients may accuse their spouse of being an impostor, may talk to imaginary figures in the environment, or their own reflection in the mirror)<br><br>(b) obsessive symptoms (e.g., continual repetition of simple cleaning activities)<br><br>(c) anxiety symptoms, agitation, and previously nonexistent violent behavior<br><br>(d) cognitive abulia (i.e., loss of willpower because they cannot carry a thought long enough to determine a purposeful course of action).
7	Very severe cognitive decline	*All verbal abilities are lost*<br><br>Frequently, there is no speech ability at all; only grunting remains.

	Patients have urinary incontinence and require assistance with toileting and eating. They lose psychomotor skills (e.g., the ability to walk). The brain appears unable to tell the body what to do. Generalized cortical and focal neurologic signs and symptoms are frequently present.

Supplementary Table 2. Pupillometer data recorded at initial and serial evaluations after carbon monoxide poisoning and categorized according to outcome groups.

	Favorable outcome (n=83, 79.8%)	Poor outcome (n=21, 20.2%)	p-value
Max. pupil size (mm)			
24-h lowest values	3.94 (3.42 – 4.65)	4.31 (3.15 – 4.76)	0.587
Total lowest values	4.65 (4.07 – 5.49)	4.70 (3.90 – 4.89)	0.619
Min. pupil size (mm)			
24-h lowest values	2.77 (2.46 – 3.18)	2.83 (2.20 – 3.51)	0.852
Total lowest values	3.09 (2.70 – 3.64)	2.98 (2.54 – 3.51)	0.644
Constriction velocity (mm/s)			
24-h lowest values	1.97 (1.54 – 2.72)	1.79 (1.30 – 2.25)	0.157
Total lowest values	1.49 (0.89 – 1.97)	0.79 (0.45 – 1.30)	0.001
Max. constriction velocity (mm/s)			
24-h lowest values	2.78 (2.24 – 3.89)	2.92 (1.79 – 3.17)	0.385
Total lowest values	2.12 (1.58 – 2.80)	1.13 (0.65 – 1.79)	0.002
Latency			
24-h lowest values	0.27 (0.23 – 0.27)	0.23 (0.23 – 0.27)	0.479
Total lowest values	0.27 (0.23 – 0.30)	0.30 (0.23 – 0.37)	0.107
Max. dilatation velocity (mm/s)			
24-h lowest values	0.94 (0.73 – 1.12)	0.72 (0.67 – 0.94)	0.032

Total lowest values	0.63 (0.41 – 0.85)	0.33 (0.21 – 0.61)	0.010

Max = maximum, Min = minimum, PLR = pupillary light reflex

Data were presented with median with interquartile intervals.

Supplementary Table 3. Comparison of the pupillometer data between patients with no sequelae vs. those with delayed neurocognitive sequelae vs. those with permanent neurocognitive sequelae.

	No sequelae N=80 (76.9%)	DNS N=16 (15.4%)	PNS N=8 (7.7%)	p-value
NPi				
24-h lowest values	4.10 (3.78 – 4.35)	4.00 (3.50 – 4.25)	3.75 (1.90 – 4.50)	0.550
Total lowest values	4.10 (3.75 – 4.30)	3.85 (3.50 – 4.15)	3.55 (1.50 – 4.35)	0.306
qPLR (%)				
24-h lowest values	21.0 (15.5 – 28.0)*	17.5 (11.5 – 26.5)	13.0 (4.5 – 18.0)*	0.038
Total lowest values	21.0 (15.5 – 27.0)*	17.5 (11.5 – 26.5)	13.0 (4.5 – 18.0)*	0.041

DNS = delayed neurocognitive sequelae, PNS = permanent neurocognitive sequelae, NPi = Neurological Pupil index, Max = maximum, Min = minimum, LR = light reflex

* indicates a difference between the two groups on the post-hoc test.

Supplementary Table 4. Comparison of the pupillometer data recorded at initial and serial evaluations after carbon monoxide poisoning between patients with no sequelae vs. those with delayed neurocognitive sequelae vs. those with permanent neurocognitive sequelae.

	No sequelae<br>N=80 (76.9%)	DNS<br>N=16 (15.4%)	PNS<br>N=8 (7.7%)	p-value
Max. pupil size (mm)				
24-h lowest values	4.65 (4.08 – 5.44)	4.79 (3.86 – 5.17)	4.70 (3.89 – 5.27)	0.967
Total lowest values	4.75 (4.22 – 5.56)	4.86 (3.99 – 5.21)	5.16 (4.07 – 5.81)	0.870
Min. pupil size (mm)				
24-h lowest values	3.09 (2.74 – 3.64)	3.19 (2.52 – 3.68)	3.07 (2.56 – 4.48)	0.955
Total lowest values	3.12 (2.78 – 3.67)	3.26 (2.56 – 3.78)	3.72 (2.74 – 5.00)	0.596
Constriction velocity (mm/s)				
24-h lowest values	1.40 (0.89 – 1.95)*	1.25 (0.71 – 1.75)	0.55 (0.27 – 0.90)*	0.007
Total lowest values	1.32 (0.88 – 1.91)*	1.25 (0.71 – 1.75)	0.55 (0.27 – 0.90)*	0.009
Max. constriction velocity (mm/s)				
24-h lowest values	2.11 (1.50 – 2.73)*	1.71 (0.96 – 2.70)	0.74 (0.63 – 1.27)*	0.012
Total lowest values	2.07 (1.37 – 2.65)*	1.71 (0.96 – 2.70)	0.73 (0.52 - 1.27)*	0.016
Latency				
24-h lowest values	0.27 (0.23 – 0.30)	0.27 (0.23 – 0.30)	0.34 (0.29 – 0.47)	0.080
Total lowest values	0.27 (0.23 – 0.30)	0.29 (0.25 – 0.33)	0.34 (0.29 – 0.47)	0.100
Max. dilatation velocity (mm/s)				
24-h lowest values	0.64 (0.41 – 0.85)[*]	0.56 (0.33 – 0.75)[#]	0.19 (0.10 – 0.26)[*#]	<0.001
Total lowest values	0.62 (0.40 – 0.85)[*]	0.56 (0.33 – 0.75)[#]	0.14 (0.07 – 0.26)[*#]	<0.001
Standard LR reactivity				0.114

Reactive	78 (97.5)	15 (93.8)	7 (87.5)
Sluggish	0 (0.0)	1 (6.3)	1 (12.5)
Non-reactive	2 (2.5)	0 (0.0)	0 (0.0)

DNS = delayed neurocognitive sequelae, PNS = permanent neurocognitive sequelae, Max = maximum, Min = minimum, PLR = pupillary light reflex.

*# indicates a difference between the two groups on the post-hoc test.
